# Favourable Short- to Mid-Term Outcome after PDA-Stenting in Duct-Dependent Pulmonary Circulation

**DOI:** 10.3390/ijerph191912794

**Published:** 2022-10-06

**Authors:** Regina Wespi, Alessia Callegari, Daniel Quandt, Jana Logoteta, Michael von Rhein, Oliver Kretschmar, Walter Knirsch

**Affiliations:** 1 Pediatric Cardiology, Pediatric Heart Center, Department of Surgery, University Children’s Hospital Zurich, 8032 Zurich, Switzerland; 2 University of Zurich, 8006 Zurich, Switzerland; 3 Child Development Center, University Children’s Hospital Zurich, 8032 Zurich, Switzerland

**Keywords:** PDA stenting, univentricular heart, duct-dependent pulmonary circulation, pediatric cardiac interventions

## Abstract

Background. Stenting of patent ductus arteriosus (PDA) is a minimally invasive catheter-based temporary palliative procedure that is an alternative to a surgical shunt in neonates with duct-dependent pulmonary perfusion. Methods. An observational, single-centre, cross-sectional study of patients with duct-dependent pulmonary perfusion undergoing PDA-stenting as a stage I procedure and an analysis of short- to mid-term follow-up until a subsequent surgical procedure (stage II), with a focus on the interstage course. Results. Twenty-six patients were treated with PDA-stenting at a median (IQR) age of 7 (4–10) days; 10/26 patients (38.5%) (6/10 single pulmonary perfusion) were intended for later univentricular palliation, 16/26 patients (61.5%) (13/16 single pulmonary perfusion) for biventricular repair. PDA diameter was 2.7 (1.8–3.2) mm, stent diameter 3.5 (3.5–4.0) mm. Immediate procedural success was 88.5%. The procedure was aborted, switching to immediate surgery after stent embolisation, malposition or pulmonary coarctation in three patients (each n = 1). During mid-term follow-up, one patient needed an additional surgical shunt due to severe cyanosis, while five patients underwent successful catheter re-intervention 27 (17–30) days after PDA-stenting due to pulmonary hypo- (*n* = 4) or hyperperfusion (*n* = 1). Interstage mortality was 8.6% (2/23), both in-hospital and non-procedure-related. LPA grew significantly (*p* = 0.06) between PDA-stenting and last follow-up prior to subsequent surgical procedure (*p* = 0.06). RPA *Z*-scores remained similar (*p* = 0.22). The subsequent surgical procedure was performed at a median age of 106 (76.5–125) days. Conclusions. PDA-stenting is a feasible, safe treatment option, with the need for interdisciplinary decision-making beforehand and surgical backup afterwards. It allows adequate body and pulmonary vessel growth for subsequent surgical procedures. Factors determining the individual patient’s course should be identified in larger prospective studies.

## 1. Introduction

Almost 20% of children with complex types of congenital heart disease (CHD) have duct-dependent pulmonary perfusion after birth [1], with a need for intravenous prostaglandin E1 (PGE1) to ensure the patency of ductus arteriosus [2,3,4,5]. Within days after birth, neonatal cardiac surgery needs to be performed [1] with a systemic-to-pulmonary shunt insertion, e.g., a modified Blalock–Taussig (mBTS) shunt, to secure pulmonary perfusion for a longer period of time until the subsequent surgical procedure [6,7,8]. However, such palliation is still associated with relevant perioperative morbidity and increased interstage mortality [9,10].

Therefore, catheter-guided patent ductus arteriosus (PDA) stent implantation has been developed as a treatment alternative. After its first description in 1992 [11], the early results of PDA-stenting were limited due to technical aspects [6,12], which have been optimised by technical modifications regarding the type of stents and soft wires as well as the use of long sheaths. In several centres worldwide, PDA-stenting has become a routine procedure with good short-term results [2,12,13,14,15], serving as an alternative treatment to systemic-to-pulmonary shunt surgery [9,16].

Despite the promising short-term results of PDA-stenting, less is known about the mid-term follow-up until the subsequent surgical procedure, including clinical course, interstage morbidity, need for readmission or re-intervention by catheter or surgery, and somatic and neurological outcomes after PDA-stenting. Furthermore, little is known about the impact of PDA-stenting on pulmonary artery development and the differences in treating duct-dependent single or biventricular CHD [17,18,19].

Therefore, this study aims to investigate the short- to mid-term outcomes of neonates with cyanotic CHD undergoing PDA-stenting as first palliation (stage I) in our institution.

## 2. Methods

### 2.1. Study Design

Within this observational, single-centre, cross-sectional follow-up study, we evaluated infants undergoing neonatal PDA stent implantation in complex types of CHD with duct-dependent pulmonary circulation between January 2013 and June 2020. In our institution, the decision for PDA-stenting is made in the daily interdisciplinary rounds upon approval by a pediatric cardiac surgeon, cardiologist, anaesthetist, and intensivist. Based on the clinical charts and catheter protocols, we analysed clinical variables and the *short-term* outcomes of PDA-stenting, including the need for surgical or catheter-guided re-interventions during follow-up. For *mid-term* outcomes after PDA-stenting, we investigated interstage mortality and morbidity determined by length of ICU and hospital stay, type and frequency of complications, somatic development determined by body weight gain and pulmonary artery growth, and neurological outcome assessed with Bayley Scales III at one year of age. Primary study endpoints were defined as elective or immediate surgical PDA stent explantation or peri-/post-procedural death before the subsequent surgical procedure (stage II).

### 2.2. PDA-Stenting

PDA-stenting, as described by Schranz et al. [15] and Alwi and Mood [6], was performed under general anaesthesia following the standard protocol for cardiac catheterisation procedures at our institution. Before or at the start of cardiac catheterisation, PGE1 was stopped according to the size of the PDA measured by transthoracic echocardiography the day before. Interventional management of PDA-stenting was dependent on duct anatomy/morphology. Duct morphology was determined by aortic angiographies in various radiographic views and classified according to a tortuosity index recently published by Qureshi et al. as type I (straight), type II (one turn), and type III (multiple turns) [20]. The stenting procedure was performed according to the duct morphology, either via the femoral venous (antegrade) route or the femoral arterial or axillary route (retrograde), using 4 French (long) sheaths. By routine, we used single bare metal coronary artery stents for complete coverage of the entire PDA length. In some cases with long and tortuous PDA morphology, additional stents were implanted to ensure complete coverage. We avoided significant stent protrusion into the neighbouring vessel structures at the aortic and pulmonary artery ends. Stent diameter was chosen according to the body weight of the neonate, expected time of palliation, and actual PDA diameter. The stent size was mostly comparable to the size of a surgical systemic-to-pulmonary shunt (3.5 mm). Peri-procedural aortic angiographies were performed through the long sheath to rule out incomplete coverage of the PDA as well as potential stent-related pulmonary artery branch stenosis. Peri-procedural anticoagulation (heparin bolus of 100 IU/kg) and antibiotic prophylaxis (cefazolin 25 mg/kg) were given at the start of the procedure. Anticoagulation continued after catheterisation using subcutaneous low-molecular-weight heparin (1.5 mg/kg every 12 h for at least 36–48 h) after successful intervention and was switched with a short overlap to oral acetylsalicylic acid monotherapy (3–5 mg per kg per day) until subsequent surgery.

### 2.3. Medical Variables

We analysed the medical data of all patients from birth until the subsequent surgical procedure following PDA-stenting, including cardiac diagnosis, genetic comorbidity, dose of PGE1, transcutaneous oxygen saturation (tcSO_2_), anticoagulation therapy, catheter procedure, PDA morphology, surgical follow-up, length of ICU and hospital stay, and interstage morbidity/mortality. Intra- and post-procedural complications were analysed. Somatic growth determined by weight-for-age *Z*-scores (WAZ) was measured at birth, at PDA-stenting, at last follow-up during subsequent cardiac catheterisation, and before the study endpoint with the subsequent surgical procedure. Pulmonary artery growth was evaluated by angiography at the time of PDA- stenting and at last follow-up prior to stage II. Pulmonary artery size was assessed using the *Z*-scores of the right and left pulmonary arteries [21,22].

Neurodevelopmental follow-up was performed initially at the age of 12 months and consecutively by clinical picture and/or routine follow-up at 24 months. The neurodevelopmental assessment included an assessment by the Bayley Scales of Infant and Toddler Development, Third Edition (Bayley-III) and a neurological exam. Bayley-III assesses different developmental domains (the cognitive composite score (CCS), the language composite score (LCS) and the motor composite score (MCS), with a mean score of 100 and a standard deviation of ±15). Later assessments also included the Griffith score and the Snijders–Oomen nonverbal intelligence test (SON-R). Neurodevelopmental outcome was rated *normal* if no neurological abnormalities were present and developmental quotient (developmental age x 100/chronological age) and/or cognitive test scores were higher than 85, *mildly impaired* if mild neurological abnormalities were present and/or developmental quotient or cognitive scores were between 70 and 85, *moderately impaired* if moderate neurological abnormalities were present or developmental quotient or cognitive scores were below 70 and *severely impaired* if severe neurological abnormalities were present and developmental quotient or cognitive scores were below 70.

### 2.4. Statistics

Statistical analysis was performed using SPSS 25 (SPSS Inc, IBM Company, Chicago Illinois/USA). Normally distributed data are presented as mean and SD, non-normal distributed data as median and interquartile range (IQR). Data are displayed by boxplot and Kaplan–Meier curves. A group comparison was performed using two-sample t-tests or Mann–Whitney tests, depending on the data distribution. Levene’s test for equality of variance was used to analyse if the variability in the two groups was significantly different. Statistical significance was set by *p* < 0.05.

The study was conducted in accordance with the declaration of Helsinki (revision 2013). The cantonal ethical committee approved the study (KEK-ZH 2020-01103).

## 3. Results

### 3.1. Patients and Cardiac Diagnoses

We included 26 patients undergoing PDA-stenting as the first palliative procedure. Patients’ characteristics and cardiac diagnosis are shown in Table 1. Median (IQR) age at PDA-stenting was 7 (4–10) days, body weight 3.3 (2.9–3.6) kg. Seventeen (65.4%) patients were male. Six patients (23.1%) had an associated genetic syndrome, including microdeletion syndrome 22q11 (*n* = 3; 11.5%). Four patients (15.4%) were pre-term infants. The most frequent cardiac diagnoses, independent of univentricular palliation or biventricular repair, were pulmonary atresia (PA) with ventricular septal defect (VSD) (*n* = 10; 38.5%), tetralogy of Fallot (TOF) (*n* = 5; 19.2%), and PA with intact ventricular septum (*n* = 5; 19.2%).

### 3.2. Management of Intravenous Prostaglandin E1

After birth, patients received PGE1, with a median (IQR) dose of PGE1 of 7.25 (4–8) ng per kg per min. In 13 patients, PGE1 was stopped 2 to 10 h before or at the start of cardiac catheterisation (*n* = 10). Three patients were not on PGE1 before cardiac catheterisation (*n* = 3); in one patient with complex PA and MAPCAs, PGE1 was stopped 30 days prior to intervention due to PGE1-independent anatomy/morphology. In two patients with isolation of the left pulmonary artery, no PGE1 was used.

### 3.3. Short-Term Follow-Up

#### 3.3.1. PDA-Stenting

Before PDA-stenting, tcSO_2_ was 86% (83–91%). According to angiographic delineation, PDA morphology was classified as type I (*n* = 9; 34.6%), type II (*n* = 8; 30.8%), or type III (*n* = 9; 34.6%). PDA diameter and length were 2.7 (1.8–3.2) and 18 (14–20) mm, respectively.

Stent implantation was performed by retrograde access via femoral artery (*n* = 18; 69.2%) or axillary artery (*n* = 3; 11.5%) or by antegrade access via femoral vein (*n* = 5; 19.2%). Eight (30.8%) patients received one stent, ten patients (38.5%) two stents, six patients (23.1%) three stents, and two patients (7.7%) four stents. The stent diameter was 3.5 (3.5–4.0) mm. Stent types used were the Multi-Link Vision^®^ stent (Abbott Vascular, Abbott Park, IL, USA) (*n* = 37; 68.5%), the Palmaz Blue^®^ stent (Cordis, Miami Lakes, FL, USA) (*n* = 11; 20.4%), the Kaname^®^ coronary stent (Terumo Corporation, Tokyo, Japan) (*n* = 4; 7.4%), and the Resolute Onyx^®^ DES stent (Dublin, Ireland) (*n* = 2; 3.7%). Procedural time was 82 (57–98) min, X-ray time 21 (11–31) min, and x-ray dose 0.7 (0.5–1.2) mcGy/m^2^.

#### 3.3.2. Stent Complications

In 3 of 26 (11.5%) patients, stent implantation was attempted but aborted due to intraprocedural stent embolisation into the left pulmonary artery (LPA) (*n* = 1) due to suboptimal PDA stent position with cyanosis (*n* = 1) and LPA stenosis (“pulmonary coarctation”) (*n* = 1). In these three patients, a subsequent surgical procedure was performed immediately, including implantation of a central aorto-pulmonary shunt with patch enlargement of the pulmonary artery bifurcation (*n* = 1), mBTS insertion with patch enlargement of the right pulmonary artery (RPA) (*n* = 1), and right ventricular outflow tract (RVOT) patch enlargement (*n* = 1) to ensure antegrade pulmonary perfusion. The postoperative and further clinical course of these three patients was uneventful.

Successful PDA-stenting was achieved in 23/26 (88.5%) patients. Minor complications were femoral arterial or venous thrombosis after catheterisation (*n* = 5) and arterio-venous fistulas (treated by pressure bandage) (*n* = 1). With anticoagulation using subcutaneous low-molecular-weight heparin, complete relief of thrombosis could be achieved in 3/5 (60%) cases. Both patients with LPA isolation and preceding reduced antegrade pulmonary perfusion showed temporary reperfusion syndrome of the left lung after PDA-stenting.

### 3.4. Mid-Term Follow-Up

#### 3.4.1. Interstage Period

**Figure 1:**
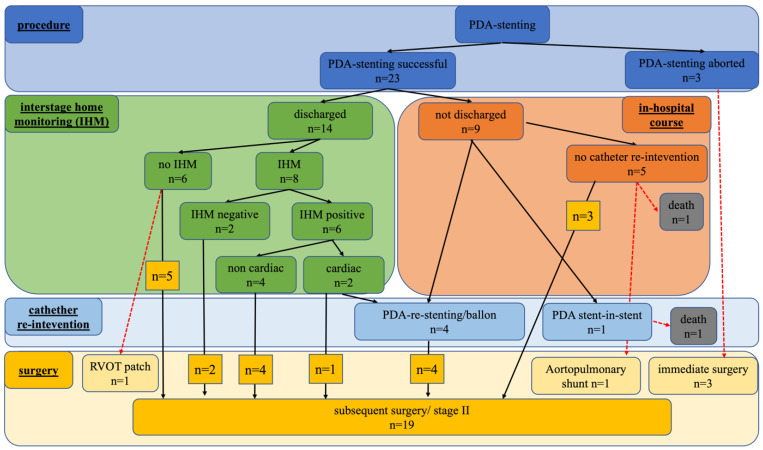
Clinical course during interstage home monitoring (IHM) (highlighted in green) or in hospital (highlighted in orange) after neonatal PDA-stenting in the duct-dependent complex type of CHD with uneventful course or with the need for emergent surgical procedures (highlighted with red arrows) or catheter re-interventions (highlighted in light blue) up to the study endpoint with the subsequent surgical procedure—stage II (highlighted in yellow).

Figure 1 shows the further clinical course over the interstage period up to the subsequent surgical procedure (study endpoint). Early discharge from the hospital was possible in 14/23 (60.9%) patients at 14 (11–17) days after PDA-stenting, with a post-procedural ICU stay of 1 (1–2) day and a total pre- and post-procedural length of hospitalisation stay of 19 (16–38) days. There were no significant differences between the discharged and not-discharged patients, as shown in Table 2. Interstage monitoring (including tcSO_2_ measurement and body weight measurement once a day) was performed in 8/14 (57.1%) patients, 7 of them with univentricular physiology and 2 of them with double supply pulmonary perfusion. A total of 6/14 (42.8%) patients were discharged without interstage monitoring, 3/6 (50%) of them with double supply pulmonary perfusion, and 3/6 (50%) with isolation of left pulmonary artery. Interstage monitoring was performed for 10 (3.3–14) weeks. No patient died at home during interstage surveillance, but 6 of 14 (42.9%) patients were positive during interstage monitoring due to respiratory infection (*n* = 4) or cyanosis (*n* = 2), leading to catheter re-intervention (*n* = 1) or elective early bidirectional cavopulmonary (Glenn) anastomosis (*n* = 1). Overall, the re-hospitalisation rate was 7/14 (50%).

One patient received an RVOT patch enlargement 61 days after PDA-stenting. This patient lived with the PDA stent and surgical RVOT enlargement (later secondary enlarged by RVOT stent and re-dilation) until a 1.5 chamber correction was performed at the age of 19 months.

Nine of twenty-three patients (39.1%) could not be discharged due to hemodynamic instability and feeding difficulties (*n* = 7) or early in-hospital mortality (*n* = 2). One infant with an atrioventricular septal defect, transposition of the great arteries, PA, and total anomalous pulmonary venous return died 14 days after PDA-stenting due to congestive heart failure despite all treatment efforts, including extracorporeal membrane oxygenation (ECMO). The PDA stent was patent on autopsy, but signs of myocardial infarction were determined as the reason for heart failure. One further patient with PA/VSD died 52 days after PDA-stenting due to severe respiratory distress syndrome, but no autopsy was performed.

#### 3.4.2. Catheter Re-Interventions

**Figure 2:**
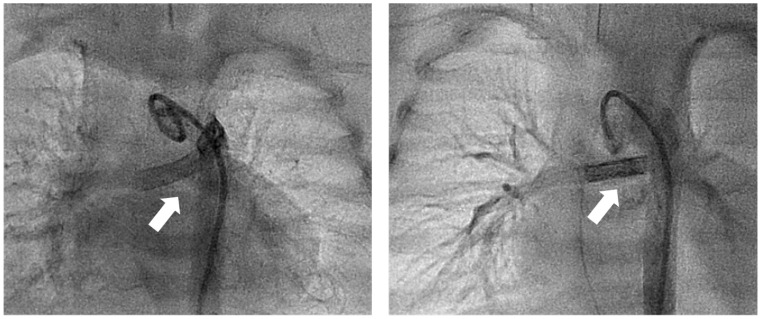
Downsizing of PDA stent. Frontal view, angiography of aortic arch showing stented PDA (white arrow) after birth at day 4 (**left**) and after re-intervention at day 17 (**right**). At day 4 (**left**), two coronary stents (Multi-Link Vision^®^, 3.5 mm diameter) were implanted. At day 17, according to a mismatch of pulmonary hyperperfusion, with clinical signs of necrotising enterocolitis, five further coronary stents were implanted, reducing the PDA stent diameter from 3.3 to 2.8 mm.

Catheter re-interventions were performed 27 (17–30) days after PDA-stenting in 5 of 23 (21.7%) patients. All re-interventions were performed in patients with a tortuosity index higher than I (2 patients with tortuosity index II and 3 with tortuosity index III), but this difference was not statistically significant (*p* = 0.2). In 4 of 5 (80%) patients, the reason for re-intervention was a restrictive PDA, leading to severe cyanosis and pulmonary hypoperfusion, treated by a secondary PDA stent (*n* = 2), PDA stent balloon dilatation (*n* = 1), or both (*n* = 1), followed by an increase of oxygen saturation (tcSO2 before 80 ± 7% vs. cSO2 after 86 ± 5%, *p* = 0.07). In 1 of 5 (20%) patients, re-intervention was performed due to pulmonary hyperperfusion with high tcSO2 (90%), high blood pressure amplitude (lowest diastolic pressure 23 to 25 mmHg), and clinical signs of necrotising enterocolitis. PDA stent diameter was reduced by stent-in-stent implantations (with five coronary stents) to decrease the stent diameter from 3.3–3.5 to 2.8–3 mm (Figure 2). Postinterventional oxygen saturation decreased (tcSO2 86%), while diastolic blood pressure increased (28–30 mmHg).

#### 3.4.3. Somatic and Pulmonary Artery Growth

Body weight growth was determined and compared using WAZ. Body weight at birth was 3.3 (2.9–3.6) kg with resp. WAZ −0.4 (−0.9 to +0.2), at time of PDA-stenting 3.3 (2.9–3.6) kg with resp. WAZ −0.7 (−1.3 to +0.1), and at subsequent surgical procedure (study endpoint) 5.5 (4.9–6.2) kg with resp. WAZ −1.6 (−1.8 to −1.0).

**Figure 3:**
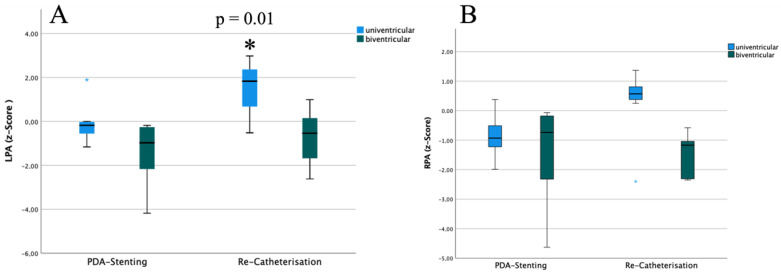
Pulmonary artery growth: Box-and-whiskers plot illustrating pulmonary artery growth; there was better LPA growth in patients undergoing later univentricular palliation compared to those with a biventricular repair. (**A**): Z-Scores for LPA at PDA-stenting and last follow-up (*p* = 0.06). Z-score LPA at last follow-up before univentricular palliation was 1.83 (+0.68 to +2.37) vs. those before biventricular repair −0.54 (−1.45 to +0.03) (*p* = 0.01). (**B**): Z-score for RPA at PDA-stenting and last follow-up (*p* = 0.22). * *p* = 0.01.

Overall pulmonary artery dimensions in the angiographies at PDA-stenting and at last follow-up were compared. *Z*-scores for LPA were *−0.39 (−1.05 to −0.18)* at PDA-stenting and *0.15 (−0.52 to +1.83)* at last follow-up (*p* = 0.06). *Z*-score for RPA was *−0.84 (−1.47 to −0.28)* at PDA-stenting and *−0.17 (−1.46 to +0.6)* at last follow-up (*p* = 0.22). The comparison between infants undergoing later univentricular palliation and those with biventricular repair is in Figure 3. In addition, LPA growth was better in patients with double supply pulmonary perfusion (*n* = 3) (Z-Score LPA at last follow-up before next surgical step *2.64 (+2.03 to 2.81)* than in patients with single pulmonary perfusion *−0.2 (−0.69 to 0.78)*).

#### 3.4.4. Subsequent Surgical Procedures

**Figure 4:**
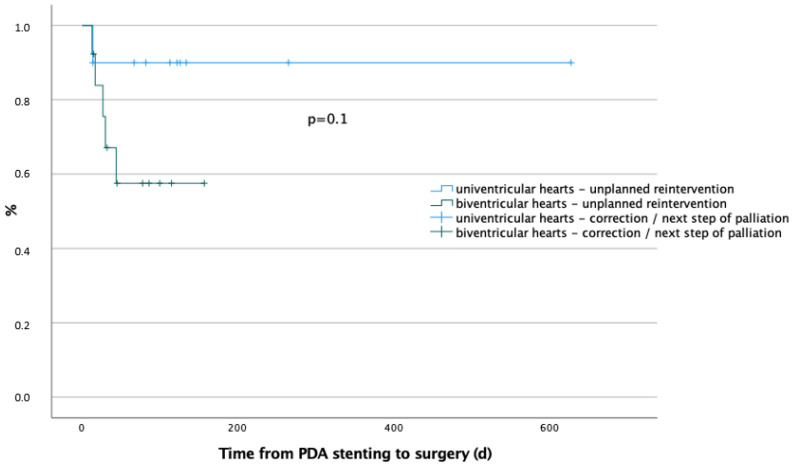
Kaplan–Meier curve comparing freedom from re-intervention in anticipated univentricular (blue) or biventricular (green) physiology (*p* = 0.1). There was no difference between the two groups.

A total of 19/23 (82.6%) patients reached the study endpoint with an elective planned surgical procedure at 106 (76.5–125) days. The implanted stents remained patent for 96 (67.5–121) days. In patients undergoing univentricular palliation, the implanted stents remained in place for 122 days (82–134) and in those with biventricular repairs, 82 (42–104) days. The difference between these two groups is shown in Figure 4.

At the time of surgery, the implanted stents could be removed or transected without any difficulties and/or any specific complications. A surgical reconstruction of the pulmonary arteries was performed in 7 patients: in 6/7 (85%) of the LPA and in 1/7 (15%) of the RPA. A total of 5/7 (70%) patients had a univentricular heart.

#### 3.4.5. Neurodevelopmental Outcome and Head Circumference

Neurodevelopmental outcome and head circumference data were available for 13 of 19 (68.4%) patients evaluated after PDA-stenting and the subsequent surgical procedure at the age of 15 (10–46) months. Overall, neurodevelopmental (both cognitive and motor) outcomes were normal to mildly impaired for both the single ventricle and biventricular patients, and severe neurodevelopmental impairment was an exception (Table 3).

## 4. Discussion

Nowadays, for patients with duct-dependent pulmonary circulation, three different treatment strategies as stage I procedures after birth are available. First, the surgical shunt procedure, such as mBTS, has been the standard of care for decades, with its well-established modifications offering excellent long-time historical experience [3,8]. Second, long-term use of PGE1 infusions may be considered in some specific situations, such as late pre-terms (32–36 weeks of gestation) and fetal-growth-restricted neonates (<2000 g) [5,23]. Third, neonatal PDA-stenting seems a feasible and safe treatment option in the case of duct-dependent pulmonary perfusion. However, both these discussed treatment strategies—the surgical shunt procedure as well as continuing PGE1 infusions—have limitations with regard to perioperative and interstage morbidity, 10% mortality as a shunt procedure complication [3,7,8,10,24,25] and the need for vascular access and long-term hospitalisation, attributed to PGE1 infusions [5,23].

Therefore, PDA-stenting may serve as a minimally invasive short-term alternative procedure [2,11,26,27] with the potential of reducing morbidity and mortality [23,25]. In fact, recent reviews have demonstrated that PDA-stenting is associated with the advantages of early survival, improved early hemodynamics and overall less morbidity compared to mBTS procedures [3,25]. In detail, PDA-stenting reduces the length of hospital stay and feeding difficulties [28], improves somatic and pulmonary artery growth until the next surgical step [3] and avoids the potential damage of the phrenic or vagal nerve and the chylothorax and the development of surgical adhesions with regard to future surgical procedures [2,9,11,12,17,26,27].

Our small clinical observational study can confirm some aspects of these findings. In fact, the overall procedural success rate of PDA-stenting in our patient cohort was high (88.5%), with a need for a short period of ICU care afterwards and possible hospital discharge after 2 to 3 weeks (60.9%).

Further, there was no interstage home mortality until the subsequent surgical procedure, and a normal or at least only mild-to-moderately impaired neurodevelopmental (both cognitive and motor) outcome on mid-term follow-up at one year of age in most of the children (Table 3) is a reasonably good result in this severely sick group of high-risk patients.

On the other hand, a total of 9 of 23 (39.1%) patients could *not be discharged* from hospital after PDA-stenting. The reason was clinical instability, with the need to stay in the ICU and the need for inotropic support, ventilation, oxygen supplementation, nutrition, and closer monitoring. The individual patient-related risk factors for infants not to be discharged after PDA-stenting could not be identified in our small study but may be independent of the different treatment strategies of PDA-stenting, the mBTS procedure, or long-term PGE1 infusion. Individual patient-related risk factors may interplay with anatomic factors of pulmonary artery hypoplasia, genetic factors, and hemodynamic factors [2,11,26,27]. In this group of non-discharged patients, there were two cases of early in-hospital mortality. One neonate died due to cardiac decompensation (on autopsy with myocardial infarction) at day 14 after PDA-stenting. The PDA stent diameter in this child was at the upper size limit (4 mm minimal size, body weight 3.8 kg), which emphasises the difficulties in balancing pulmonary to systemic blood flow, resulting in low diastolic blood pressure with a risk for pulmonary blood run-off, also well-known after surgical shunt procedures [3].

The technical aspects of PDA-stenting regarding PDA morphology, the use of long sheaths, the need for multiple stents and the antegrade or retrograde approach may be underlined. Even tortuous PDA morphology, often more challenging for PDA-stenting due to the risk of ductal spasms [29], is treated with increasing experience with comparable results [9,20], including pre-terms with low birth weight [12,30]. Some aspects are crucial for a successful short- to mid-term result [15]: the use of long sheaths suitable for a stable stent delivery [9], careful decision-making for the optimal retrograde or antegrade approach, depending on the PDA course [31,32] and the necessity for complete stent coverage of the straightened PDA. Therefore, in some patients, up to four separate stents per patient were necessary for complete coverage of the PDA; the implantation of further PDA stents may be more challenging due to the risk of stent dislocation [33], as experienced in one case with stent dislocation of the left pulmonary artery. In some specific anatomic situations, an axillary vascular approach was used, allowing straight access to the PDA [34].

Procedural complications of PDA-stenting were rare, in line with the results of recent reports [9,25,35]. Of note, all these technical problems were handled with surgical backup, which from our point of view, is a major key factor for collaboration leading to no peri-procedural deaths. This highlights the fundamental need for interdisciplinary decision-making and surgical backup to perform PDA-stenting safely. Other severe adverse events did not occur [24,25,29].

Re-interventions were mostly performed (21.7%) with PDA-re-stenting or re-ballooning [36], as described in the literature [25]. In our cohort, we only had one case of acute stent stenosis with an early need for re-intervention after PDA-stenting [29]. Acute shunt thrombosis has been described for both surgical shunts and PDA stents, but with a higher incidence in surgical shunts (in up to 9% of patients). Glatz et al., in a multi-centre study, did not find a significant difference regarding death or unplanned re-intervention as a primary endpoint when comparing PDA-stenting and the surgical mBTS procedure. However, Bentham et al. reported a higher re-intervention rate when comparing PDA-stenting (39.8%) and surgical mBTS procedures (24%) [3]. In our cohort, re-interventions were mostly (*n* = 4) due to pulmonary hypoperfusion with low oxygen saturation levels. In one patient, pulmonary perfusion could be reduced by stent-in-stent implantation.

As described by Shahanavaz et al. [37], all re-interventions were performed in patients with more tortuous duct morphology. A possible explanation lies in the abnormal flow patterns that could result in neointima proliferation and in the additional procedural complexity imposed by a torturous arterial duct.

However, in comparison to Shahanvaz et al. [37], who reported a higher incidence of re-interventions for patients with anticipated univentricular physiology, in our cohort, the incidence of re-interventions was higher in patients with biventricular physiology (Figure 4). This is probably because in patients with univentricular anatomy, the implanted stents remained longer in place.

PDA stent removal or transection during subsequent surgery was always feasible, without complications and independently of the time interval between implantation and surgery [2,25]. Furthermore, the lack of surgical scars deriving from previous surgeries reduced the technical difficulty of the subsequent surgery.

Pulmonary artery growth is induced by PDA-stenting as well as by surgical shunt procedures [12,35,37]; PDA-stenting is favoured, with more balanced pulmonary vascular development [12]. This may be explained by the PDA stent being in a more “natural” position or angle [12,19] compared to the flow pattern of an aortic-to-pulmonary shunt, as recently shown by Emarsafawy and coworkers [19]. On the other hand, the RPA is limited in growth development as the blood flow through the stented PDA favours the LPA [3,6,7,24,25]. Our data confirm these findings. In our cohort, patients with univentricular anatomy showed better pulmonary artery growth than patients scheduled for biventricular repair, which may be partly explained by the intrinsic hypoplasia of pulmonary arteries in patients with PA and VSD or with TOF (Figure 3).

The influence of PDA-stenting on body growth has been assessed before [28]. Our data are in line with previously published results, and our cohort showed adequate somatic growth up to the subsequent surgical step. Re-hospitalisation due to feeding difficulties or failure to gain weight never occurred. Regarding neurological outcomes, most of our patients showed a normal to mildly impaired neurodevelopmental cognitive outcome, and severe neurodevelopmental impairment was an exception. Our findings regarding neurological outcomes are comparable with the results of other research groups as well as the results of our own research group “Heart and Brain” [38,39].

### Limitations

The limited number of cases and the short to mid-term follow-up period, together with the retrospective design, are the main limitations of this study. Furthermore, a comparison with patients with other approaches (e.g., mBTS) has not been performed so far. Due to the small patient cohort, the influence of single/double supply pulmonary perfusion on the growth of the pulmonary arteries could not be evaluated. Moreover, we only assessed the short- to mid-term follow-up. Whether PDA-stenting requires additional surgical manoeuvres on the pulmonary arteries or further interventions during long-term follow-up has not been evaluated.

## 5. Conclusions

In neonates with ductal-dependent pulmonary circulation, PDA-stenting is a feasible, safe, and successful alternative treatment to the classical surgical shunt procedure as a stage I procedure. The interdisciplinary team approach offers an individual patient process to determine factors for optimised pulmonary and somatic growth as well as neurodevelopmental outcomes.

## Figures and Tables

**Table 1:** Study population.

Patients (*n*=, %)	26 (100)
Age at PDA-stenting (days)	7 (4–10)
Male (*n*=)	17 (65)
Weight (kg)	3.3 (2.9–3.6)
Body surface area (m^2^)	0.21 (0.20–0.23)
Gestational age (weeks)	38.3 (37.6–39.0)
Pre-term (*n*=)	4 (15)
Cardiac diagnosis (*n*=, %)	
*Univentricular CHD*	10 (38)
Pulmonary atresia with VSD (*n*=)	2 (8)
Pulmonary atresia with intact ventricular septum (*n*=)	5 (19)
Tricuspid atresia (*n*=)	1 (4)
Ebstein anomaly (*n*=)	1 (4)
Double outlet right ventricle (VSD type) (*n*=)	1 (4)
*Biventricular CHD*	16 (62)
Tetralogy of Fallot (*n*=)	5 (19)
Pulmonary atresia with VSD (*n*=)	7 (27)
Pulmonary atresia with VSD and aorto-pulmonary collaterals (*n*=)	1 (4)
Left pulmonary artery isolation (*n*=)	2 (8)
Double outlet right ventricle (Fallot type) (*n*=)	1 (4)
Pulmonary perfusion (*n*=, %)	
Single supply	19 (73)
Double supply	7 (27)

Data presented as median (IQR), categorial data as counts (n) and percentages (%). CHD, congenital heart disease; VSD, ventricular septal defect.

**Table 2:** Comparison of discharged and non-discharged infants after PDA-stenting during short- to mid-term follow-up until subsequent surgical procedure.

	Discharged (*n* = 14)	Non-Discharged (*n* = 9)	*p*
Sex (male)	12/14 (86)	4/9 (45)	**0.04**
Gestational age (w)	38.5 (37.4–39.4)	38.1 (37.6–38.6)	0.62
Prematurity (<37 weeks) (n)	3/14 (21)	0/9 (0)	0.15
Birth weight (kg)	3.2 (2.9–3.4)	3.1 (3.0–3.3)	0.51
Body surface area	0.21 (0.19–0.21)	0.20 (0.19–0.21)	0.99
Single pulmonary perfusion (n)	9/14 (64)	7/9 (78)	0.52
Genetic syndrome (n)	3/14 (21)	3/9 (33)	0.55
Age (d) at stent implantation	7.5 (6–13)	6 (4–10)	0.39
Weight (kg) at stent implantation	3.3 (3–3.7)	3.3 (3–3.5)	0.49
Length (cm) at stent implantation	50 (48–52)	49 (48–51)	0.39
Procedural time (min)	88 (56–99)	73 (62–85)	0.92
X-ray time (min)	18.5 (11.3–26.1)	22 (16–32)	0.62
X-ray exposure (G/cm^2^)	0.71 (0.43–1.28)	0.7 (0.6–0.9)	0.66
PGE1 before intervention (n)	10/14 (71)	9/9 (100)	0.08
Oxygen saturation before intervention (%)	87 (84–92)	85 (83–88)	0.46
Oxygen saturation after intervention (%)	91 (87–94)	90 (85–90)	0.48
Stents used pro patients (n)	2 (1–3)	2 (1–2)	0.32
Ductal tortuosity index:			
Tortuosity Index Typ I (n)	4/14 (28)	3/9 (33)	0.82
Tortuosity Index Typ II (n)	5/14 (36)	2/9 (22)	0.52
Tortuosity Index Typ III (n)	5/14 (36)	4/9 (44)	0.69
Typical duct anatomy (n)	10/14 (71)	7/9 (78)	0.75
Atypical duct anatomy (n)	4/14 (29)	2/9 (22)	0.75
Thrombosis after intervention(n)	2/14 (14)	3/9 (33)	0.30
Anticoagulation:			
Change to ASS possible (n)	14/14 (100)	7/9 (78)	0.07
Change to ASS after n days (n)	3.5 (1–6.25)	1 (1–9.5)	0.85
ICU time (d)	1 (1–3)	1 (1–2)	0.89
NEC (y/n)			
Bell Stage II:	1/14 (7)	1/9 (11)	0.75
Bell Stage III:	0/14 (0)	1/9 (11)	0.22
Mortality (n)	0/14 (0)	2/9 (22)	0.07
Total hospitalisation time (d)	18.5 (16–34)		
Unplanned surgery (n)	0/14 (0)	1/9 (11)	0.75
Interstage (n)	8/14 (57)		
Interstage mortality (n)	0/14 (0)		
Total interstage time (w)	10 (3.3–14)		
Re-interventions (n)	1/14 (7)	4/9 (44)	**0.04**
Time period until surgery (d)	118 (89.5–132)	45 (18.5–73)	0.08
Univentricular palliation (n)	6/14 (43)	1/7 (7)	0.29
1.5 chamber correction (n)	2/14 (14)	0/7 (0)	0.30
Biventricular correction (n)	6/14 (43)	6/7 (86)	0.09
Body weight growth (g/d)	22.6 (20.7–27.2)	24 (18–27.5)	0.89

Data are presented as median (IQR), categorial data as counts (n) and percentages (%). D: days; w: weeks; g: gram. Bold: significant *p*-value. y: yes; n: no.

**Table 3:** Head circumference and neurodevelopmental outcome of infants with complex types of CHD evaluated after primary PDA-stenting and subsequent surgical palliation for univentricular CHD (stage II) or surgical repair for biventricular CHD for short- to mid-term outcomes.

Age (Months)	Head Circumference (Percentile)	Neuromotor Findings	Cognitive Assessment Tool	Cognitive Development Quotient or BSID III CCS	Sum Score
***Univentricular CHD after primary PDA-stenting***					
19	>97	No pathologies	BSID III	85	0
46	50–75	Mild hypotonia	SON 2–8	63	2
26	10–25	No pathologies	Griffith	85	0
22	10–25	No pathologies	BSID III	50	2
10	3–10	Mild hypotonia	BSID III	100	1
***Biventricular CHD after primary PDA-stenting***					
14	50–75	No pathologies	BSID III	100	0
15	<3	No pathologies	BSID III	55	2
13	10–25	Mild hypotonia	BSID III	60	2
15	no data	CP	BSID III	60	3
21	50–75	No pathologies	BSID III	100	0
11	3–10	Motor delay (BSID III MCS: 79)	BSID III	105	1
12	25–50	No pathologies	BSID III	115	0
11	<3	Motor delay (BSID III MCS: 61)	BSID III	70	3

The sum score was calculated according to neurological impairment, and the calculated developmental quotient was defined as normal (0): no neurological impairment and developmental quotient >85; mildly impaired (1): mild neurological abnormalities and/or developmental quotient 70–85; moderately impaired (2): moderate neurological abnormalities or developmental quotient <70; severely impaired (3): severe neurological abnormalities and/or developmental quotient <70. BSID III, Bayley Scales of Infant Development III; CCS, cognitive composite score, MCS, motor composite score. CP, cerebral paralysis; SON 2-8, SON-R 2-8 nonverbal intelligence test.

## Data Availability

The authors confirm that the data supporting the findings of this study are available within the article. Supplementary data that support the findings of this study are available from the corresponding author, W.K., upon reasonable request.
